# Efficacy and safety of fractional microablative radiofrequency versus topical estriol in treating genitourinary syndrome of menopause: A pilot study

**DOI:** 10.1002/ijgo.70563

**Published:** 2025-10-09

**Authors:** Priscila de Almeida Torre, Susana Cristina Aidé Viviani Fialho, Isabel Cristina Chulvis do Val Guimarães, Ana Ximena Zunino, Tuani Castro, Caroline Alves de Oliveira Martins, Ana K. Gonçalves

**Affiliations:** ^1^ Department of Obstetrics and Gynecology Federal Fluminense University (UFF) Rio de Janeiro RJ Brazil; ^2^ Health Sciences Center Federal University of Rio Grande do Norte (UFRN) Natal RN Brazil

**Keywords:** estriol, menopause, radiofrequency, treatment

## Abstract

**Objective:**

To compare the efficacy and safety of fractional microablative radiofrequency (RF) and topical estriol in the treatment of genitourinary syndrome of menopause (GSM).

**Methods:**

This pilot randomized controlled trial included 30 healthy postmenopausal women with GSM. Participants were randomly assigned to receive either fractional microablative RF plus placebo cream (RF group) or topical estriol plus sham RF (estriol group). Both treatments were administered over 3 months. Primary outcomes included vaginal health (Vaginal Health Index Score—VHIS), sexual function (Female Sexual Function Index—FSFI), and urinary symptoms (International Consultation on Incontinence Questionnaire—Short Form—ICIQ‐SF). Safety was assessed through reports of adverse effects.

**Results:**

Both RF and estriol significantly improved VHIS, FSFI, and ICIQ‐SF scores (*p* < 0.05). Estriol provided greater improvement in sexual desire, while RF demonstrated superior results in satisfaction, dyspareunia reduction, and epithelial elasticity. No severe adverse effects occurred; mild discomfort was reported in the RF group.

**Conclusion:**

Fractional microablative RF and topical estriol are both effective and safe for relieving GSM symptoms. RF offers a promising non‐hormonal alternative, especially for women who cannot or choose not to use estrogen therapy.

Genitourinary syndrome of menopause (GSM) affects up to 84% of postmenopausal women and tends to worsen over time if left untreated. Common symptoms include vaginal dryness, dyspareunia, dysuria, urgency, urinary incontinence, and recurrent infections. Despite its high prevalence, approximately 70% of affected women remain untreated or unaware of effective therapies. While local estrogen therapy remains the gold standard, emerging alternatives such as laser and radiofrequency (RF) treatments are gaining attention.^1^, ^2^, ^3^, ^4^

This study compared the efficacy and safety of fractional microablative RF and topical estriol in treating GSM. Key outcomes included vaginal health, sexual function, urinary incontinence, and treatment tolerability.

A pilot randomized controlled trial (No. 55925422.5.00005243) was approved by the research ethics committee of the Faculty of Medicine at the Federal University of Rio de Janeiro. Thirty postmenopausal women diagnosed with GSM were enrolled. Participants were eligible if they were healthy, sexually active, postmenopausal women (follicle‐stimulating hormone > 40 mIU/mL; estradiol < 25 pg/mL), with vulvovaginal atrophy (Vaginal Health Index ≤ 15), normal cervical smear, and negative HPV test. Exclusion criteria included recent use of hormonal therapies, lubricants or vaginal moisturizers, genital infections, vulvar dermatological disorders, and precancerous or painful conditions.

Eligible participants provided informed consent and were randomly assigned (via random.org) to one of two double‐blinded groups: the RF group (treated with RF and placebo vaginal cream) and the estrogen group (treated with topical estriol and placebo RF pulses [sham]).

Both groups underwent three RF sessions (one per month, spaced 25–40 days apart). The topical treatment involved a 21‐day consecutive application of estriol followed by three weekly applications for 3 months. Placebo creams were used in the same regimen to maintain blinding.

Outcomes were assessed using the Vaginal Health Index Score (VHIS), Female Sexual Function Index (FSFI), and International Consultation on Incontinence Questionnaire – Short Form (ICIQ‐SF). Safety was evaluated using the visual analog scale for pain, edema, and exudation.

Participants completed the FSFI and ICIQ‐SF questionnaires, followed by a blinded gynecological examination to assess vaginal health (VHIS) and pH. All assessments were repeated at the study's conclusion.

Adverse events such as pain, edema, exudation, and bleeding were evaluated before each intervention, 30 days after each session, and at the end of the treatment.

The participants' mean ± standard deviation age was 56.33 ± 7.10 years (range, 42 to 69 years), and the mean ± standard deviation age at menopause onset was 44.70 ± 5.74 years. The average time since menopause was 10 years, indicating that most participants had experienced a significant period of estrogen deprivation.

The VHIS score increased significantly after treatment in both groups, demonstrating overall improvement in the vaginal health of the participants (*P* < 0.05). These improvements included vaginal moisture: RF (*P* < 0.001) and estrogen (*P* = 0.002) groups; vaginal fluid volume: RF and estrogen groups (*P* < 0.001); vaginal pH: RF (*P* = 0.039) and estrogen (*P* < 0.001) groups; vaginal elasticity: RF (*P* = 0.038) and estrogen (*P* < 0.001) groups; and vaginal epithelial integrity: RF and estrogen groups (*P* < 0.001) (Table 1).

**TABLE 1:** Evaluations of VHIS, FSFI, and ICQ‐SF.

VHIS				
Variable	Group	Before treatment (mean ± SD)	After treatment (mean ± SD)	*P* value
Vaginal moisture	RF	2.33 ± 1.11	3.53 ± 1.06	<0.001
	Estrogen	2.67 ± 1.11	3.93 ± 0.70	0.002
Vaginal fluid volume	RF	1.80 ± 1.10	2.93 ± 0.96	<0.001
	Estrogen	2.20 ± 1.08	3.60 ± 0.83	<0.001
Vaginal pH	RF	2.13 ± 1.30	3.13 ± 1.46	0.039
	Estrogen	2.47 ± 1.30	3.80 ± 0.56	<0.001
Vaginal elasticity	RF	2.53 ± 1.25	3.47 ± 1.25	0.038
	Estrogen	2.47 ± 0.91	3.80 ± 0.77	<0.001
Vaginal epithelial integrity	RF	2.87 ± 1.46	4.27 ± 0.70	<0.001
	Estrogen	3.00 ± 1.60	4.87 ± 0.35	<0.001

FSFI				
Variable	Group	Before treatment (median [Q1; Q3])	After treatment (median [Q1; Q3])	*P* value
Desire	RF	2.40 [1.20; 2.40]	3.60 [2.40; 4.20]	0.007
	Estrogen	2.92 ± 1.38	4.20 ± 0.99	<0.001
Arousal	RF	0.30 [0.00; 1.80]	3.90 [2.70; 5.10]	0.003
	Estrogen	2.70 [0.60; 3.90]	4.50 [2.10; 4.50]	0.020
Lubrication	RF	0.30 [0.00; 2.70]	3.60 [2.70; 4.80]	0.005
	Estrogen	3.00 [0.30; 4.20]	5.10 [2.40; 5.70]	0.006
Orgasm	RF	0.00 [0.00; 2.00]	3.60 [1.20; 5.20]	0.006
	Estrogen	3.60 [0.00; 4.40]	5.20 [1.20; 5.60]	0.029
Satisfaction	RF	2.59 ± 1.33	3.73 ± 1.35	0.002
	Estrogen	2.80 [1.60; 6.00]	5.60 [2.40; 6.00]	0.026
Pain	RF	0.00 [0.00; 1.60]	4.80 [1.20; 5.60]	0.003
	Estrogen	2.00 [0.80; 4.00]	5.20 [4.40; 6.00]	0.003
Global FSFI	RF	7.10 [3.80; 15.10]	24.30 [17.50; 29.00]	0.001
	Estrogen	17.40 [6.30; 22.90]	29.60 [15.10; 32.30]	0.003

ICIQ‐SF			
Group	Before (median [Q1; Q3])	After (median [Q1; Q3])	*P* value
RF	6.00 [0.00; 10.00]	0.00 [0.00; 0.00]	0.005
Estrogen	6.00 [0.00; 15.00]	3.00 [0.00; 5.00]	0.012

*Note*: Significant difference: *P* < 0.05.

Abbreviations: FSFI, Female Sexual Function Index; ICIQ‐SF, International Consultation on Incontinence Questionnaire – Short Form; Q, quartile; RF, (fractional microablative) radiofrequency; VHIS, Vaginal Health Index Score.

In addition, we found improvement in the FSFI scores for both groups in all domains, including FSFI Global: RF (*P* = 0.001) and estrogen (*P* = 0.001) groups. The estrogen group showed greater improvement in desire (*P* < 0.001), while the RF group had better outcomes in satisfaction (*P* = 0.002) and dyspareunia reduction (*P* = 0.003) (Table 1).

Urinary complaints decreased in both groups following treatment. In the estrogen group, the median ICIQ‐SF score decreased from a median of 6.00 (range, 0.00–15.00) before treatment to 3.00 (range, 0.00–5.00) after treatment (*P* = 0.012). The ICIQ‐SF score in the RF group decreased from a median of 6.00 (range, 0.00–10.00) to 3.00 (range, 0.00–0.00) after treatment (*P* = 0.005). No serious adverse effects were reported. The RF group experienced mild pain, mostly near the vaginal introitus (Table 1).

These findings align with those from Sarmento et al., who found that RF and estrogen had similar effectiveness in improving sexual function and vaginal health. Both treatments significantly outperformed the control group, with no notable differences.^5^

In addition, de Moraes et al.^6^ found that among RF, vaginal estrogen, and moisturizers, only RF significantly improved sexual desire, arousal, and orgasm in postmenopausal women. RF was deemed the most effective treatment, although long‐term results require further study.^6^

Conclusion: We found that both RF and topical estriol effectively alleviated GSM symptoms. RF offered distinct benefits in enhancing epithelial integrity and elasticity, positioning it as a promising nonhormonal alternative for women who cannot^7^ or choose not to use hormone therapy.

## AUTHOR CONTRIBUTIONS

P.A.T. was responsible for the study project and design, acquiring the location, analysis and interpretation of data, and writing and proofreading of the article. S.C.A.V.F., I.C.C.V.G., C.A.O.M., T.C., and A.X.Z. contributed to the data capture and revision of the article. A.K.G. contributed to the study design, the design and interpretation of data, and writing of the article.

## CONFLICT OF INTEREST STATEMENT

The authors declare no conflicts of interest.

## INFORMED CONSENT

Consent was obtained from the patient, and the authors maintain the document.

## Data Availability

Data sharing is not applicable to this article as no new data were created or analyzed in this study.
